# The study of epigenetic clocks in former professional contact sports athletes with repetitive head impacts

**DOI:** 10.1136/jnnp-2025-338206

**Published:** 2026-05-11

**Authors:** Xuelin Tang, Vishaal Sumra, Christine Sato, Danielle Moreno, Mozhgan Khodadadi, Nusrat Sadia, Carmen Wong, Brenda Colella, Robin E A Green, Janet Ta, Kaj Blennow, Henrik Zetterberg, Richard Wennberg, Charles H Tator, Ming Zhang, Ekaterina Rogaeva, Maria Carmela Tartaglia

**Affiliations:** 1 Tanz Centre for Research in Neurodegenerative Diseases, University of Toronto, Toronto, Ontario, Canada; 2 Department of Neurology, Neurology and Neurological Rehabilitation Center, Yangzhi Rehabilitation Hospital (Shanghai Sunshine Rehabilitation Centre), Clinical Center for Brain and Spinal Cord Research, School of Medicine, Tongji University, Shanghai, China; 3 State Key Laboratory of Cardiology and Medical Innovation Center, Shanghai East Hospital, School of Medicine, Tongji University, Shanghai, China; 4 Institute of Medical Sciences, University of Toronto, Toronto, Ontario, Canada; 5 Canadian Concussion Centre, Toronto Western Hospital, Toronto, Ontario, Canada; 6 Institute of Neuroscience and Physiology, Department of Psychiatry and Neurochemistry, The Sahlgrenska Academy at the University of Gothenburg, Mölndal, Sweden; 7 Clinical Neurochemistry Laboratory, Sahlgrenska University Hospital, Mölndal, Sweden; 8 UK Dementia Research Institute, UCL, London, UK; 9 Department of Neurodegenerative Disease, UCL, London, UK; 10 Hong Kong Center for Neurodegenerative Diseases, Clear Water Bay, China; 11 Department of Pathology and Laboratory Medicine, University of Wisconsin School of Medicine and Public Health, Madison, Wisconsin, USA; 12 Wisconsin Alzheimer’s Disease Research Center, University of Wisconsin School of Medicine and Public Health, Madison, Wisconson, USA; 13 Centre for Brain Research, Indian Institute of Science, Banglore, India; 14 Institute for Advanced Study, Tongji University, Shanghai, China; 15 Toronto Dementia Research Alliance (TDRA), Toronto, Ontario, Canada

**Keywords:** CONCUSSION, HEAD INJURY, TRAUMATIC BRAIN INJURY, MRI, GENETICS

## Abstract

**Background:**

Repetitive head impacts in former contact sport athletes are associated with cognitive impairment, accelerated cerebral atrophy and risk of neurodegenerative disease. Epigenetic clocks derived from age-associated DNA methylation (DNAm) profiles may capture accelerated biological ageing in neurodegenerative conditions; however, their application in former athletes remains unexplored. Here we aim to explore the application of epigenetic clocks as a measure of accelerated biological ageing in a cohort of former athletes.

**Methods:**

In 126 former athletes (96% male; mean age: 54.5±14.4 years; mean concussions: 6.8±6.7), we examined associations of brain volumes, plasma neurofilament light levels and cognitive/behavioural scores with DNAmAge-acceleration, AgeAccelResidual and DNAmFitAge-acceleration.

**Results:**

We only found an association between the number of concussions and DNAmFitAge-acceleration (p=0.003, B=0.46, R²=0.063), indicating that every two additional concussions were associated with a 5-year increase in DNAmFitAge-acceleration. There was also a trend towards an association between years of play and DNAmAge-acceleration in older athletes.

**Conclusions:**

These preliminary findings suggest that specific epigenetic clock measures may serve as early markers of biological ageing related to repetitive head impacts.

**WHAT IS ALREADY KNOWN ON THIS TOPIC**

The authors reviewed the literature using traditional sources (eg, PubMed) and meeting abstracts. Though DNA methylation studies have investigated age acceleration in neurodegenerative diseases, there is a gap in the research when examining the effects of repeated head trauma on accelerated ageing. These relevant citations are appropriately cited.

**WHAT THIS STUDY ADDS**

Our findings suggest that repeated head impacts are associated with accelerated epigenetic ageing, which may be age-dependent.

**HOW THIS STUDY MIGHT AFFECT RESEARCH, PRACTICE OR POLICY**

The manuscript proposes a framework for the generation of new hypotheses and the conduct of additional studies. Examples include further understanding: (a) the relationship between years of play and accelerated ageing; (b) the influence of proximity to play (ie, years since professional career) and accelerated ageing and (c) the relationship between the number of concussions, neuroinflammatory changes and accelerated ageing.

## Background

 Repetitive head impacts (RHI) in contact sports have been implicated in neurodegenerative diseases, including chronic traumatic encephalopathy (CTE), Parkinson’s disease, Alzheimer’s disease and amyotrophic lateral sclerosis.^1–3^ There is increasing evidence that RHI are associated with behavioural changes, cognitive deficits and accelerated brain atrophy, whereas normal ageing is associated with only mild brain volume loss accompanied by minor cognitive deficits.^4 5^ We have previously shown that a group of retired Canadian Football League players had poorer verbal memory performance and greater hippocampal atrophy than expected for their age.^5^ Others have confirmed this finding.^6^ Such observations could be the result of accelerated ageing, as has been reported in connection with diabetes or with abnormal amyloid levels in cerebrospinal fluid.^7^

Recently, there is accumulating evidence that chronological age does not sufficiently represent fundamental ageing processes and that biological and chronological age are discrepant.^8^ Methods to measure biological ageing have become available and may provide novel insights into factors impacting ageing as well as neurodegeneration. Specifically, the effects on ageing of various environmental and lifestyle factors (eg, physical activity) should be evaluated.

The most investigated markers of ageing are based on the DNA methylation (DNAm) of cytosines at CpG dinucleotides, representing one of the key epigenetic mechanisms altering gene expression or exon splicing.^8^ DNAm levels at specific CpG sites can be shaped by environmental factors and life experiences, such as RHI. To assess biological ageing, which captures interindividual differences in ageing, several epigenetic clocks have been developed based on age-related DNAm profiles.

The most studied epigenetic clock is the multi-tissue Horvath’s DNAmAge, as it maintains a consistent tick rate across most tissues, including blood and brain.^9^ DNAmAge is derived from the collective assessment of ~350 CpGs and provides several metrics, including DNAmAge-acceleration, defined as the deviation of DNAmAge from chronological age, reflecting the raw difference but remaining correlated with chronological age. Another common metric is AgeAccelResidual, the residual from a linear regression model, which captures the deviation from the expected DNAmAge after adjusting for chronological age. In addition, DNAmAge metrics can be affected by differences in cell type composition, race and sex.^10^

A study of twin cohorts suggested that non-genetic factors become increasingly significant with age. For instance, the broad-sense heritability of DNAmAge-acceleration is ~100% in newborns but decreases to ~39% in older individuals.^9^ DNAm-age-acceleration has been associated with an earlier onset of several neurodegenerative diseases,^8^ including rapid eye movement sleep behaviour disorder, which is the strongest prodromal marker for α-synucleinopathies (eg, Parkinson’s disease).^11^ Notably, such patients tend to be epigenetically older than controls, further supporting a connection between accelerated ageing and prodromal neurodegeneration.^12^

There is emerging evidence that epigenetic clocks are influenced by lifestyle factors^13–16^ and that even individual differences in fitness parameters are reflected in DNAm data.^14 15^ For instance, the DNAmAge could be slowed by 3.2 years following an 8-week diet and lifestyle intervention (eg, exercise).^17^ Currently, there are no reports addressing the connection between epigenetic clocks and brain ageing in former contact sports athletes exposed to RHI.

Given that individuals with RHI can develop prodromal neurodegeneration, the current study explored the relationship between epigenetic age and various clinical measures, MRI measures of brain volume and plasma neurofilament light chain (NfL), a biomarker of neurodegeneration, in former contact sports athletes with RHI. We evaluated Horvath’s DNAmAge, along with a new epigenetic clock (DNAmFitAge) that incorporates physical fitness.^18^ Each of these epigenetic measures could offer unique insights into ageing mechanisms.

## Methods

### Participants

We evaluated 126 former professional (n=104) and semiprofessional (n=22) contact sports athletes with RHI enrolled in a longitudinal concussion monitoring programme at the Canadian Concussion Centre, University Health Network, Toronto, Canada. Table 1 presents the characteristics of the cohort, which consisted of 96% males, with a mean age at baseline of 54.5±14.4 years and a mean number of concussions of 6.8±6.7, ranging from 0 to 35. Sports played included American football, hockey, boxing, soccer, volleyball, snowboarding, basketball and polo. Data on the number of years played—from childhood to the end of the professional career—were available for 114 former athletes. Longitudinal DNAm assessments were available for 21 former athletes who donated blood up to three times over a period of 1–10 years (table 1).

**Table 1:** Sample characteristics of the former contact sports athletes

Sample characteristics	Baseline N (%) or mean (SD)	Follow-up N (%) or mean (SD)
Samples, N	126	21
Age at sample collection, years	54.52 (14.37)	Baseline: 54.62 (13.18) follow-up: 59.83 (12.32)
Sex, N (%)		
Male	121 (96.03%)	21 (100%)
Female	5 (3.97%)	0 (0.00%)
Race, N (%)		
White	112 (88.88%)	17 (80.95%)
Black	10 (7.94%)	2 (9.52%)
Arab	2 (1.59%)	2 (9.52%)
Other (Hispanic, West Asian)	2 (1.59%)	0 (0.00%)
Number of concussions	6.78 (6.75)	6.90 (7.39)
Years played (n=114)	16.59 (6.74)	
Sport, N (%)		
American football	83 (65.87%)	
Hockey	28 (22.22%)	
Soccer	2 (1.58%)	
Basketball	1 (0.79%)	
Volleyball	1 (0.79%)	
Polo	1 (0.79%)	
Snowboarding	1 (0.79%)	
Professional, N (%)	104 (82.54%)	
Semiprofessional, N (%)	22 (17.46%)	
Plasma NfL (n=96)	11.38 (7.13)	Follow-up (n=12): 30.44 (68.21)
Executive Composite Score	0.23 (0.74)	Baseline: 0.26 (0.58)
Memory Composite Score	−0.13 (1.07)	Baseline: −0.22 (1.11)
Mood Composite Score	0.16 (0.85)	Baseline: −0.11 (0.76)
Horvath DNAmAge, years	61.35 (13.59)	Baseline: 62.04 (11.67) follow-up: 66.50 (11.7)
DNAmFitAge, years	55.04 (14.48)	Baseline: 55.08 (12.67) follow-up: 61.06 (12.18)

DNAm, DNA methylation; NfL, neurofilament light chain.

All participants underwent a semistructured interview where concussion exposure was determined based on the player’s recall of injury, using the concussion definition provided by the Concussion in Sport Group.^19^ Exclusion criteria comprised neurological disorders predating concussions (eg, seizure disorders), systemic illnesses known to affect the brain (eg, lupus) and a history of psychotic or developmental disorders. Informed consent was obtained from all participants, and the study was approved by the University Health Network Research Ethics Board.

### Cognitive and mood/behavioural composite scores

All participants underwent a neuropsychological assessment, including mood and personality testing by a trained psychometrist supervised by a psychologist using normed, standardised measures. Since multiple concussions are associated with cognitive decline (eg, executive dysfunction and memory deficits) and neuropsychiatric alterations (eg, depression, apathy, impulsivity and aggression),^5 20^ we created three composite scores (executive function, memory function and mood/behaviour) by averaging individual z-scores of each test from the neuropsychological assessment. The executive function composite score was created by averaging the z-scores of the following measures: Digit Span Backward, Paced Auditory Serial Addition Test^21^ and phonemic verbal fluency scores. The memory function composite score was created by averaging the z-scores of the following measures: Rey Auditory Verbal Learning Test total learning, short delay recall and long delay recall. Finally, the mood/behaviour composite score was derived by averaging z-scores from the Personality Assessment Inventory:^22^ Anxiety, Depression, Mania and the Aggression treatment considerations scale that includes aggression (with subscales: aggressive attitudes, verbal aggression and physical aggression).

### Evaluation of NfL levels in blood plasma

Plasma NfL levels were available for 96 retired athletes, as a subset of participants consented to blood draws for genetic analysis only. NfL concentration was measured using single-molecule array (Simoa) technology and the commercial NF-Light assay (Quanterix, Billerica, Massachusetts). Samples were diluted two-fold with assay diluent and analysed as singlicates. Quality control included analysis of duplicate samples, and only the runs with intra-assay and interassay coefficient of variation <15% were included.

## MRI acquisition

All 126 participants were scanned using a high-resolution T1-weighted MRI scan. Prior to 2023, 103 subjects were scanned using a T1-weighted inversion recovery prepped, 3-dimensional IR-FSPGR (inversion fast spoiled gradient echo) sequence at 3 Tesla (GE Signa HDx, Milwaukee, Wisconsin, USA) with the following parameters: echo time=3.0 ms, repetition time=7.8 ms, inversion time=450 ms, flip angle=158°, resolution=1 mm^3^ isotropic. The remaining 23 subjects were scanned using an axial 3D T1-weighted magnetization-prepared rapid gradient echo (MPRAGE) sequence that was acquired in a 3 Tesla Siemens MAGNETOM Prisma Scanner, with the following parameters: echo time=3.7 ms, inversion time=1.1 s, repetition time=2400 ms, resolution=1 mm^3^ isotropic, flip angle=8°. No difference in signal-to-noise ratio was observed between the two scanners.

### Brain volume evaluation

#### Grey matter volume and cortical thickness analysis

T1-weighted images were processed using the recon-all command in FreeSurfer (v7.4) to generate grey matter volumes and cortical thickness. Final segmented grey matter volumes from left and right hemisphere cortical grey matter regions in the FreeSurfer (Desikan-Killiany) atlas were corrected for intracranial volume.

#### Voxel-based morphometry: voxelwise analysis

For voxel-based morphometry analysis, we used the Statistical Parametric Mapping (SPM12) toolbox.^23^ T1-weighted structural MRIs were segmented into grey matter, white matter and cerebrospinal fluid volumes. Grey matter volume maps were then registered to Montreal Neurological Institute template space and smoothed using an 8 mm full-width half-maximum kernel.

### DNAm analyses

All DNA samples were processed at the Microarray Facility of the Centre for Applied Genomics (Toronto, Canada). Age-related DNAm levels were measured using the genome-wide Infinium MethylationEPIC chip (Illumina, USA), which accommodates up to eight samples per chip. To minimise batch effects, all 126 samples were processed together at the same time. The DNAm analyses were conducted mainly as previously reported.^11^ In brief, genomic DNA was extracted from blood samples using a QIAGEN kit (Hilden, Germany) and stored at −20°C until bisulfite conversion using the EZ DNA Methylation-Lightning Kit (Zymo Research, Orange, California). DNA sample storage time ranged from 0.5 to 11 years.

To obtain DNAmFitAge,^18^ DNAmAge and AgeAccelResidual,^9^ the raw DNAm data was analysed by the Horvath calculator (https://dnamage.clockfoundation.org/). We focused on DNAmFitAge, as it integrates epigenetic health and physical fitness, making it particularly relevant for our cohort of former athletes. Horvath’s DNAmAge was prioritised due to its stable tick rate across most tissues, including blood and brain, aligning with the brain measures assessed in this study. We calculated DNAmAge-acceleration and DNAmFitAge-acceleration by subtracting the chronological age at sample collection from the DNAmAge and DNAmFitAge, respectively. Notably, DNAmFitAge is constructed separately for males and females^18^ and is derived from DNAm-based proxies of fitness-related biomarkers, including CpGs that automatically track biological sex using the Horvath calculator.

### Statistical analyses

We conducted multivariate linear regression analyses to assess the associations between DNAmFitAge-acceleration, DNAmAge-acceleration or AgeAccelResidual with NfL plasma levels, cortical brain volumes using data from the FreeSurfer segmentation. Regions of interest from the FreeSurfer atlas include the left and right cortical grey matter. All statistical analyses were performed using R (V.4.1.0), with a significance threshold set at an adjusted p value of <0.05 controlling for chronological age except for AgeAccelResidual, which is, by its design, already correlated with chronological age. We included chronological age as a covariate in regression models to control potential residual confounding by age because previous studies have shown that epigenetic age acceleration metrics may retain a modest correlation with chronological age.^9^ In addition, the results were adjusted for DNA storage time and epigenetic estimates of blood cell composition (CD8T, CD4T, B cells, granulocytes, natural killer cells and monocytes). Benjamini–Hochberg false discovery rate (FDR) correction for multiple comparisons (k=15) was applied.

We also conducted stratified analysis focusing on one sex (male) or race (White), the most prevalent groups in the cohort (table 1). Notably, males have been reported to show a slightly higher Horvath epigenetic age than females of the same chronological age,^10^ and a similar pattern has been observed for DNAmFitAge.^18^ However, our results are unlikely to be influenced by sex differences, as the cohort consisted predominantly of males. To examine whether the five female participants were outliers, we applied Tukey’s 1.5×IQR range criterion. None of the female participants were identified as outliers for any of the investigated epigenetic measures. Female values fell within the outlier thresholds for DNAmFitAge-acceleration (−0.30 to 6.85; thresholds −9.61 to 10.20), DNAmAge-acceleration (−2.10 to 7.67; thresholds −4.64 to 18.5) and AgeAccelResidual (−9.46 to 0.83; thresholds −10.6 to 10.8).

To assess the relationship between epigenetic clock measures and grey matter volume on a whole-brain level, the smoothed-spatially normalised grey matter probability maps resulting from voxel-based morphometry processing were assessed via the general linear model, controlling for age and total intracranial volume.

## Results

### Chronological age is linked with brain volumes, cortical thickness and NfL plasma levels

**Figure 1:**
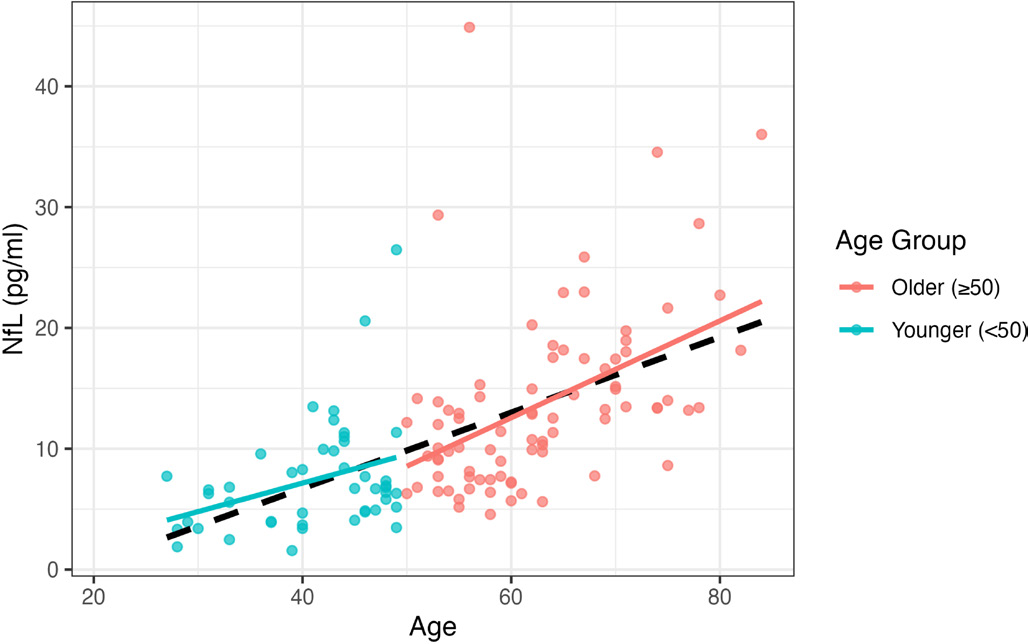
Scatter plot of chronological age versus NfL plasma levels, which were available for the 96 former athletes. NfL, neurofilament light chain.

Investigated cohort included 126 former athletes (table 1), a part of which (n=53) has been comprehensively described elsewhere.^5^ In agreement with prior observations, multivariate linear regression analyses of the enlarged cohort revealed a significant association of chronological age with both grey matter volume and cortical thickness in the left (p_volume_=2.62×10^-10^, p_thickness_=0.01) and right (p_volume_=2.08×10^-10^, p_thickness_=0.01) hemispheres. NfL plasma levels were also strongly positively associated with chronological age (p=2.4×10^-12^, B=0.57, R^2^=0.33, n=96) (figure 1). In contrast, chronological age was not associated with memory (p=0.36, B=−0.05, R² = 0.02), mood (p=0.14, B=−0.01, R² = 0.01) or executive function scores (p=0.16, B=−0.01, R² = 0.02). Notably, the number of concussions was negatively related to executive function (p=0.0038, B=−0.022, R² = 0.031) but not associated with memory, mood, NfL measures or chronological age.

### Epigenetic clock measures remain stable over the follow-up period

**Figure 2:**
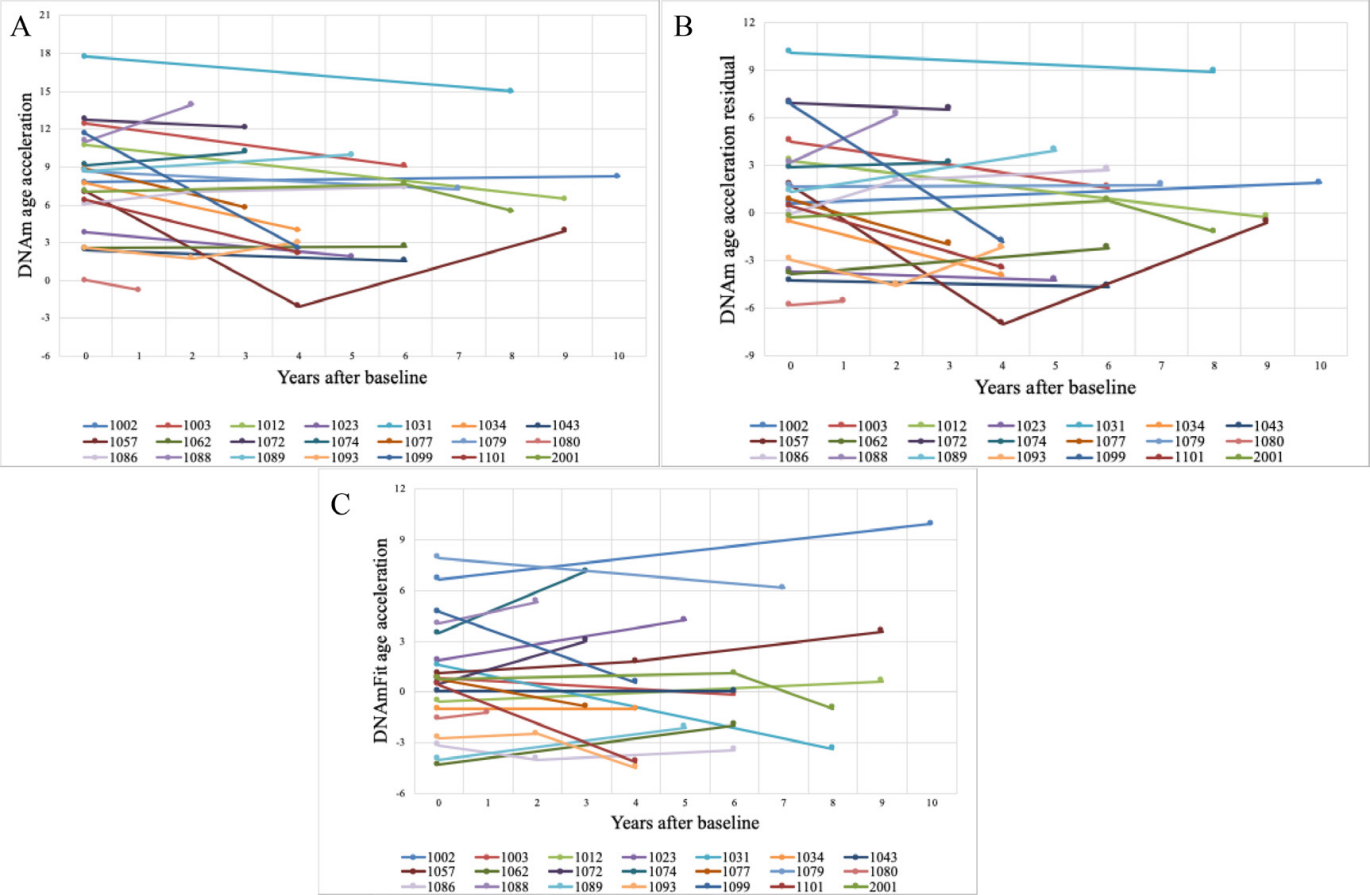
Line charts showing longitudinal changes in epigenetic clock measures over up to 10 years for 21 former athletes. Each differently coloured line represents a different athlete, with up to three time points of sample collection. (A) DNAmAge-acceleration; (B) AgeAccelResidual and (C) DNAmFitAge-acceleration. DNAm, DNA methylation.

At baseline (n=126), DNAmAge was older than chronological age in 93% of former athletes, while AgeAccelResidual and DNAmFitAge were above chronological age in only half of the subjects (online supplemental figure 1). Longitudinal assessments revealed that for most of the 21 former athletes with repeated blood draws, all three measures of epigenetic age acceleration remained steady for up to 10 years (figure 2). Most subjects in the longitudinal cohort exhibited rising epigenetic age with advancing chronological age, and the stability of this pattern underscores the potential of epigenetic clocks as robust biomarkers of biological ageing.

### Epigenetic clock measures are not linked with NfL plasma levels, clinical measures, cortical thickness, or brain volumes

We did not detect a significant association of NfL plasma levels, memory scores, executive function or mood with DNAmAge-acceleration, AgeAccelResidual or DNAmFitAge-acceleration, after correcting for multiple comparisons. Furthermore, in the voxelwise analysis (using the general linear model in SPM12), no significant associations were observed between grey matter probability maps and the epigenetic clock measures.

### DNAmFitAge-acceleration is associated with the number of concussions

**Figure 3:**
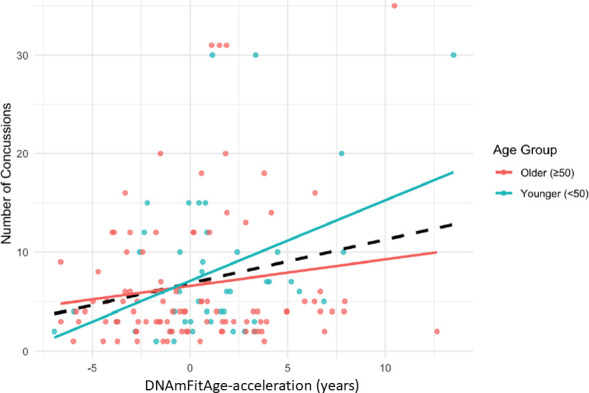
Scatter plot of DNAmFitAge-acceleration and number of concussions in former athletes (p=0.0023, B=0.46, R²=0.063, N=126), depicted by a dashed line. The positive association is mainly driven by younger former athletes (<50 years; p=0.005, B=0.40, R²=0.16, N=44), depicted by a green line. DNAm, DNA methylation.

Multivariate linear regression analysis revealed a significant positive association of DNAmFitAge-acceleration with number of concussions in former athletes (p=0.003, B=0.46, R² = 0.063), indicating that every additional two concussions corresponded to a 5-year increase in DNAmFitAge-acceleration (figure 3). This association remained significant after adjustment for DNA storage time (p=0.004, B=0.44, R²=0.111) and blood cell composition (p=0.003, B=0.51, R² = 0.070). In addition, we conducted a male-only analysis, which did not significantly affect the strength of the association (p=0.002, B=0.50, R²=0.070). Finally, we repeated the analysis restricted to White participants (table 1), and the results remained largely unchanged (p=0.006, B=0.45, R²=0.057), indicating that inclusion of non-White participants (n=12) did not affect the observed associations. Notably, all these adjustments did not alter the absence of associations with Horvath clock measures (data not shown).

When stratified by age (<50 years; n=44; and ≥50 years N=82), a significant positive association between number of concussions and DNAmFitAge-acceleration was observed only in the younger athletes (p=0.005, B=0.40, R²=0.160), suggesting an age-dependent relationship (figure 3). A similar result was observed after adjusting for age at sample collection (p=0.004, B=0.44, R²=0.06). In contrast, the measures of Horvath’s epigenetic clock (DNAmAge-acceleration and AgeAccelResidual) were not associated with the number of concussions.

### The relationship between years of play and clinical or epigenetic clock measures

Within our cohort of former athletes, multivariate linear regression revealed that the number of years played was not associated with executive function, mood, memory scores, chronological age, number of concussions, NfL levels (<50 years; n=30; ≥50 years N=55) or measures of epigenetic clocks. However, when athletes were stratified by age (≥50 years), there was a trend towards a significant positive relationship between years of play and AgeAccelResidual in the older group (p=0.099, B=0.19, R²=0.038, ≥50 years; n=73), suggesting a potential age-dependent effect between the number of years played and epigenetic age acceleration.

## Discussion

We conducted the first investigation of epigenetic clocks in former athletes with a history of RHI. DNAmAge was older than chronological age in 93% of former athletes, whereas only half of the cohort was epigenetically older based on AgeAccelResidual or DNAmFitAge-acceleration. None of the investigated epigenetic measures correlated with either plasma NfL levels, cognitive performance, brain volumes or cortical thickness. Notably, most of our participants exhibited normal cognition, showed no clinical evidence of neurodegeneration, and would be expected to be at early prodromal disease stages. Yet, we confirmed that chronological age is significantly associated with cerebral volumes and NfL plasma levels, as expected based on prior studies.^24 25^ In the future, we will continue epigenetic investigation of the same cohort in later disease stages.

Interestingly, DNAmFitAge-acceleration was significantly associated with the number of concussions, largely driven by the younger former athletes (age <50), who may be closer to a high fitness state. DNAmFitAge was created to incorporate phenotypic information to produce a novel indicator of biological age, which combines physical fitness and epigenetic health.^18^ This epigenetic clock integrates the established prediction of mortality risk (DNAmGrimAge) with the newly developed predictions of fitness. Physically fit individuals tend to have a younger DNAmFitAge and better age-related outcomes.^18^ Even though our study participants were no longer professional athletes, many retain higher activity levels, and some were still playing sports. Their physical fitness may be reflected in the smaller proportion of individuals with an advanced DNAmFitAge compared with their DNAmAge. However, the high incidence of concussions could offset the benefits associated with their physical fitness.

Notably, functional annotation of the 627 CpGs used to construct DNAmFitAge revealed enrichment in inflammation-related genes, supporting hypotheses that link physical fitness and systemic inflammation.^18^ Neuroinflammation has been implicated in the pathophysiology of neurodegeneration after RHI.^26 27^ Acute microglial activation and cytokine release that can occur after RHI transition to chronic inflammation, which can perpetuate tau phosphorylation and synaptic loss. Clusters of reactive microglia are often found in the subcortical white matter in CTE, associated with axonal swellings and distorted profiles in the subcortical U-fibres.^2^ Multiple perivascular foci of reactive microglia in cerebral white matter further emphasise the relationship between vascular structures, inflammation and the characteristic distribution of pathology in CTE.^2^ The chronic inflammatory state appears to create a self-perpetuating cycle, where neuroinflammation promotes protein aggregation and tau hyperphosphorylation, which in turn triggers further inflammation. This cycle helps explain the progressive nature of CTE even years after the cessation of RHI, as the pathological process continues independently of new injuries.^28^

Chronic inflammation is a hallmark of ageing and is associated with alterations in DNAm patterns.^29^ These epigenetic changes can disrupt normal gene expression, affecting immune responses and cellular repair mechanisms. Studies have shown that elevated levels of inflammatory markers (eg, interleukin-6, C reactive protein and tumour necrosis factor-alpha) correlate with changes in DNAm, potentially accelerating biological ageing.^30 31^

RHI is thought to affect axons mainly through rotational acceleration.^32–34^ These rotational forces can affect axons throughout the brain, even in the absence of clinically apparent concussion. There is growing concern that subconcussive (asymptomatic) impacts can still contribute to the pathological cascade when experienced repeatedly.^35^ The cumulative strain from RHI appears to be more significant than single severe injuries in the development of CTE, explaining why the condition is strongly associated with repetitive exposure rather than isolated traumatic events.

The lack of association between epigenetic age and brain volumes may be due to the heterogeneity of neurodegeneration following RHI and the varying prodromal disease stages among our former contact sports athletes. Although at least half showed accelerated epigenetic age, the effect on brain volume is complex, as alterations in functional connectivity may precede atrophy,^36^ which likely occurs at a later stage of the disease. Additionally, some of the other measures that may be associated with epigenetic age (eg, neuroinflammation) were not captured by this study. Beyond axonal damage, dendritic structures such as spines are vulnerable to concussive injury due to their cytoskeletal architecture.^37^ Studies have demonstrated that dendritic spines in hippocampal neurons and cortical regions show significant reductions shortly after traumatic brain injury.^37^ This spine loss may contribute to synaptic dysfunction and impaired connectivity between neurons, affecting network function and cognitive processes, but not reflected in brain volume or cortical thickness.

Years played have been considered a proxy for RHI and have been correlated with thalamic volumes^38^ and the severity of postmortem tau pathology.^39^ However, years played are recognised as a crude measure because they do not capture the substantial variability introduced by the type of sport, position, playing time and other factors. Although no relationship was found at the group level between years played and the investigated epigenetic clocks, a trend was observed among athletes over the age of 50, which may suggest an age-dependent relationship between years played and age-related DNAm.^38^ This is in keeping with numerous studies that report that RHI has effects on depression, cognition,^40^ white matter integrity^41^ and neurodegeneration^5^ are largely absent or subtle in most younger players but become pronounced in middle-aged and older retired players, particularly those with the highest cumulative exposure. This has been attributed to the additive burden of ageing on already compromised neural tissue, with RHI acting as a ‘primer’ whose consequences compound over time.

Our study has some limitations. First, our findings require validation in independent studies and further investigation of an appropriate control cohort (eg, former athletes who participated in non-contact sports). Second, our sample size is relatively small for an epigenetic study, although large for a study in former professional athletes with RHI. This limitation is particularly relevant to the negative findings between DNAm metrics and brain volumes, NfL, cognition and mood, as the study may have been underpowered to detect these associations. Finally, the epigenetic measures in this study were derived from blood DNA rather than brain tissue. However, it has previously been demonstrated that age-related DNAm changes are conserved across tissue.^9 42^ Additionally, age acceleration has been observed based on both blood and brain tissue in both Huntington’s disease and Down syndrome.^43 44^

## Conclusion

In this preliminary study, DNAmFitAge-acceleration was observed to be associated with the number of reported concussions. However, this result, as well as the negative findings, should be interpreted with caution, given the exploratory design and limited sample size. For instance, it remains unclear whether these findings can be generalised to female athletes or to non-White populations. As no single metric can fully capture biological ageing, examining additional epigenetic clocks in future research may provide further insight into potential links between RHI and ageing-related processes. The pathophysiology of RHI likely involves complex and interconnected mechanisms that may evolve over time, potentially contributing to delayed clinical outcomes such as those seen in CTE. Understanding how these mechanisms relate to biological ageing remains speculative at this stage, but could eventually inform strategies for identifying at-risk individuals or potential biomarkers. Larger, longitudinal studies are needed to clarify whether associations between RHI and measures of biological ageing exist and whether they are meaningful for predicting susceptibility to neurodegenerative outcomes.

## Supplementary material

10.1136/jnnp-2025-338206 online supplemental file 1

## Data Availability

Data are available upon reasonable request.

## References

[1] Omalu BI, DeKosky ST, Minster RL (2005). Chronic traumatic encephalopathy in a National Football League player. Neurosurgery.

[2] McKee AC, Stein TD, Kiernan PT (2015). The neuropathology of chronic traumatic encephalopathy. Brain Pathol.

[3] Taghdiri F, Khodadadi M, Sadia N (2024). Unusual combinations of neurodegenerative pathologies with chronic traumatic encephalopathy (CTE) complicates clinical prediction of CTE. Eur J Neurol.

[4] Cole JH, Leech R, Sharp DJ (2015). Prediction of brain age suggests accelerated atrophy after traumatic brain injury. Ann Neurol.

[5] Misquitta K, Dadar M, Tarazi A (2018). The relationship between brain atrophy and cognitive-behavioural symptoms in retired Canadian football players with multiple concussions. Neuroimage Clin.

[6] Bernick C, Shan G, Zetterberg H (2020). Longitudinal change in regional brain volumes with exposure to repetitive head impacts. Neurology (ECronicon).

[7] Uchida Y, Nishimaki K, Soldan A (2024). Acceleration of Brain Atrophy and Progression From Normal Cognition to Mild Cognitive Impairment. JAMA Netw Open.

[8] Bergsma T, Rogaeva E (2020). DNA Methylation Clocks and Their Predictive Capacity for Aging Phenotypes and Healthspan. Neurosci Insights.

[9] Horvath S (2013). DNA methylation age of human tissues and cell types. Genome Biol.

[10] Horvath S, Gurven M, Levine ME (2016). An epigenetic clock analysis of race/ethnicity, sex, and coronary heart disease. Genome Biol.

[11] Senkevich K, Pelletier A, Sato C (2023). DNA Methylation Age Acceleration as a Potential Biomarker for Early Onset of Rapid Eye Movement Sleep Behavior Disorder. Ann Neurol.

[12] Baldelli L, Pirazzini C, Sambati L (2023). Epigenetic clocks suggest accelerated aging in patients with isolated REM Sleep Behavior Disorder. NPJ Parkinsons Dis.

[13] Horvath S, Erhart W, Brosch M (2014). Obesity accelerates epigenetic aging of human liver. Proc Natl Acad Sci U S A.

[14] Soerensen M, Li W, Debrabant B (2019). Epigenome-wide exploratory study of monozygotic twins suggests differentially methylated regions to associate with hand grip strength. Biogerontology.

[15] Ferrari L, Vicenzi M, Tarantini L (2019). Effects of Physical Exercise on Endothelial Function and DNA Methylation. Int J Environ Res Public Health.

[16] Spólnicka M, Pośpiech E, Adamczyk JG (2018). Modified aging of elite athletes revealed by analysis of epigenetic age markers. Aging (Milano).

[17] Fitzgerald KN, Hodges R, Hanes D (2021). Potential reversal of epigenetic age using a diet and lifestyle intervention: a pilot randomized clinical trial. Aging (Milano).

[18] McGreevy KM, Radak Z, Torma F (2023). DNAmFitAge: biological age indicator incorporating physical fitness. Aging (Milano).

[19] McCrory P, Meeuwisse W, Dvorak J (2016). Consensus statement on concussion in sport-the 5(th)International Conference on Concussion in Sport Held in Berlin October 2016. Br J Sports Med.

[20] Goswami R, Dufort P, Tartaglia MC (2016). Frontotemporal correlates of impulsivity and machine learning in retired professional athletes with a history of multiple concussions. Brain Struct Funct.

[21] Spreen O, Strauss E (1998). A Compendium of Neuropsychological Tests: Administration, Norms, and Commentary.

[22] Morey LC (2007). Personality Assessment Inventory (PAI): Professional Manual.

[23] Gaser C, Dahnke R, Thompson PM (2024). The Alzheimer’s Disease Neuroimaging I. CAT: a computational anatomy toolbox for the analysis of structural MRI data. Gigascience.

[24] Fujita S, Mori S, Onda K (2023). Characterization of Brain Volume Changes in Aging Individuals With Normal Cognition Using Serial Magnetic Resonance Imaging. JAMA Netw Open.

[25] Karoly HC, Skrzynski CJ, Moe E (2021). Investigating Associations Between Inflammatory Biomarkers, Gray Matter, Neurofilament Light and Cognitive Performance in Healthy Older Adults. Front Aging Neurosci.

[26] Corps KN, Roth TL, McGavern DB (2015). Inflammation and neuroprotection in traumatic brain injury. JAMA Neurol.

[27] Simon DW, McGeachy MJ, Bayır H (2017). The far-reaching scope of neuroinflammation after traumatic brain injury. Nat Rev Neurol.

[28] Ruchika F, Shah S, Neupane D (2023). Understanding the Molecular Progression of Chronic Traumatic Encephalopathy in Traumatic Brain Injury, Aging and Neurodegenerative Disease. Int J Mol Sci.

[29] Baechle JJ, Chen N, Makhijani P (2023). Chronic inflammation and the hallmarks of aging. Mol Metab.

[30] Irvin MR, Aslibekyan S, Do A (2018). Metabolic and inflammatory biomarkers are associated with epigenetic aging acceleration estimates in the GOLDN study. Clin Epigenetics.

[31] Tejera CH, Zhu P, Ware EB (2025). DNA methylation age acceleration mediates the relationship between systemic inflammation and cognitive impairment. Epigenomics.

[32] Unterharnscheidt F, Higgins LS (1969). Traumatic lesions of brain and spinal cord due to nondeforming angular acceleration of the head. Tex Rep Biol Med.

[33] Gennarelli TA, Thibault LE, Adams JH (1982). Diffuse axonal injury and traumatic coma in the primate. Ann Neurol.

[34] Adams JH, Graham DI, Murray LS (1982). Diffuse axonal injury due to nonmissile head injury in humans: an analysis of 45 cases. Ann Neurol.

[35] Johnson B, Neuberger T, Gay M (2014). Effects of subconcussive head trauma on the default mode network of the brain. J Neurotrauma.

[36] Garcia-Cordero I, Vasilevskaya A, Taghdiri F (2024). Functional connectivity changes in neurodegenerative biomarker-positive athletes with repeated concussions. J Neurol.

[37] Brody DL, Benetatos J, Bennett RE (2015). The pathophysiology of repetitive concussive traumatic brain injury in experimental models; new developments and open questions. Mol Cell Neurosci.

[38] Schultz V, Stern RA, Tripodis Y (2018). Age at First Exposure to Repetitive Head Impacts Is Associated with Smaller Thalamic Volumes in Former Professional American Football Players. J Neurotrauma.

[39] Cherry JD, Tripodis Y, Alvarez VE (2016). Microglial neuroinflammation contributes to tau accumulation in chronic traumatic encephalopathy. Acta Neuropathol Commun.

[40] McAllister T, McCrea M (2017). Long-Term Cognitive and Neuropsychiatric Consequences of Repetitive Concussion and Head-Impact Exposure. J Athl Train.

[41] Alosco ML, Tripodis Y, Baucom ZH (2023). White matter hyperintensities in former American football players. Alzheimers Dement.

[42] Horvath S, Zhang Y, Langfelder P (2012). Aging effects on DNA methylation modules in human brain and blood tissue. Genome Biol.

[43] Horvath S, Langfelder P, Kwak S (2016). Huntington’s disease accelerates epigenetic aging of human brain and disrupts DNA methylation levels. Aging (Milano).

[44] Horvath S, Garagnani P, Bacalini MG (2015). Accelerated epigenetic aging in Down syndrome. Aging Cell.

