# Supplementary material for: The study of epigenetic clocks in former professional contact sports athletes with repetitive head impacts
Source: J Neurol Neurosurg Psychiatry. 2026 May 11;97(9):e338206. doi: 10.1136/jnnp-2025-338206 (PMC13479679; doi:10.1136/jnnp-2025-338206)
Supplement: Supplementary file 1 [file jnnp-97-9-s001.docx]

**Supplemental Figure 1.** Distribution of epigenetic age measures among former contact sports athletes (N = 126): **A**) DNAmAge-acceleration; **B**) AgeAccelResidual; and **C**) DNAmFitAge-acceleration.

A

B

C
